# Measurement and rapid assessment of indoor air quality at mass gathering events to assess ventilation performance and reduce aerosol transmission of SARS-CoV-2

**DOI:** 10.1177/01436244221137995

**Published:** 2022-12-16

**Authors:** Liora Malki-Epshtein, Filipa Adzic, Ben M Roberts, Elizabeth Abigail Hathway, Christopher Iddon, Murat Mustafa, Malcolm Cook

**Affiliations:** 1 Department of Civil, Environmental and Geomatic Engineering, 4919 University College London, London, UK; 2 Building Energy Research Group, 5156 Loughborough University, Loughborough, UK; 3 Department of Civil and Structural Engineering, 7315 University of Sheffield, Sheffield, UK; 4 6123 University of Nottingham, Nottingham, UK; 5 1981 University of the West of England, Bristol, UK

**Keywords:** COVID-19, indoor air quality, post-occupancy, CO_2_ monitoring, infection control

## Abstract

To assess risk factors for COVID-19 transmission and address the closure of mass gathering events since March 2020, the UK Government ran the Events Research Programme (ERP), following which it reopened live events in sports, music, and culture in July 2021. We report the rapid post-occupancy evaluation of Indoor Air Quality (IAQ) and associated long-range airborne transmission risk conducted in the Environmental Study of the ERP. Ten large venues around the UK were monitored with CO_2_ sensors at a high spatial and temporal resolution during 90 events. An IAQ Index based on CO_2_ concentration was developed, and all monitored spaces were classified in bands from A to G based on their average and maximum CO_2_ concentrations from all events. High resolution monitoring and the IAQ Index depicted the overall state of ventilation at live events, and allowed identification of issues with ventilation effectiveness and distribution, and of spaces with poor ventilation and the settings in which long-range airborne transmission risk may be increased. In numerous settings, CO_2_ concentrations were found to follow patterns relating to event management and specific occupancy of spaces around the venues. Good ventilation was observed in 90% of spaces monitored for given occupancies.

***Practical applications:*** High-resolution monitoring of indoor CO_2_ concentrations is necessary to detect the spatial variation of indoor air quality (IAQ) in large mass gathering event venues. The paper summarises COVID-19 ventilation guidance for buildings and defines a methodology for measurement and rapid assessment of IAQ during occupancy at live events that can be implemented by venue managers. Comparisons of the CO_2_ concentrations measured during the events identified the spaces at high risk of long-range transmission of airborne pathogens. Building operators should be mindful of the ventilation strategies used relative to the total occupancy in different spaces and the occupant’s activities.

## Introduction

In the course of the COVID-19 pandemic, there was a need for guidance on the operation of large buildings during mass gathering events to limit the transmission of Severe Acute Respiratory Syndrome Coronavirus 2 (SARS-CoV-2); this guidance remains necessary, still, to mitigate against future outbreaks. This was a primary concern due to several well publicised “super-spreading” events and after it was shown that mass gathering events encouraged the spread of SARS-CoV-2 due to crowding^1–4^ and due to insufficient ventilation of uncontaminated air into the space.^5,6^ However, knowledge of ventilation provision and indoor air quality data from such buildings was limited and previous studies usually did not address whether there was a risk due to lack of ventilation. Mass gathering events, which might include music festivals, theatre performances, and spectator sports, are places where large numbers of people will come into close contact. As such, the suspension of mass gathering events was an early pandemic mitigation measure and was considered especially useful before pharmaceutical countermeasures, such as vaccines, were available.^1^ It is vital, however, that mass gathering events are enabled, due to the positive contribution they make to the economy and the mental wellbeing of the populace.

Some pathogens, such as SARS-CoV-2,^7^ SARS,^8^ MERS,^9^ rhinovirus, and parainfluenza,^10^ are transmitted between people via airborne routes: either long-range (virus-laden aerosols) or short-range (a combination of virus-laden droplets and virus-laden aerosols), which emanate from the respiratory tract of infected people.^7^ SARS-CoV-2 has been shown to remain viable in air samples.^11,12^ Aerosols, due to their small size and mass (<50 µm)^13^ may stay suspended in the indoor air for several hours, moving long distances throughout the space where they can be inhaled by room occupants, before depositing or dispersing.^14^

Short-range airborne transmission occurs when an infector and susceptible person are in close proximity (1–2 m). For susceptible people sharing air in a space with an infector, long-range (i.e., beyond the range of 1–2 m) aerosol-based or, long-range airborne, transmission is possible, and this is exacerbated by poor ventilation. Ventilation, which introduces uncontaminated air into a space, is an important mechanism to disperse and dilute the concentration of aerosols in the air and with these, any potential airborne pathogens. There are several examples of “super-spreading events” of transmission occurring in poorly ventilated indoor environments, e.g., a restaurant,^15^ an apartment building,^16^ and in a church hall.^17^ Provision of sufficient ventilation is therefore a key long-range airborne transmission mitigation measure.^5^

There have been a number of studies that have explored the correlation between CO_2_ concentrations and exhaled breath and the risk of exposure to airborne diseases and this paper does not attempt to review all of these. Notably, Morawska et al.^18^ demonstrated that physiological processes such as breathing, talking, and coughing produce large numbers of very small aerosols and larger droplets in varying concentrations and Morawska and Milton^19^ speculated that these aerosols may lead to transmission by inhalation. Rudnick and Milton^20^ proposed a model of airborne infection transmission based on the rebreathed fraction, the fraction of inhaled air that has been exhaled previously by someone in the building, using CO_2_ concentration. They considered CO_2_ concentrations above outdoor values as a surrogate for exhaled breath, and thus as a marker for exhaled breath exposure, because most buildings do not contain other indoor sources of CO_2_. An attempt to correlate CO_2_ to aerosols experimentally in a real-world scenario relevant to the events sector was made by Schade et al.,^21^ who attempted to correlate aerosol dispersion and CO_2_ concentrations produced by a mannequin “breathing” realistically in an empty concert hall and have found some correlation between the two parameters, under a specific ventilation regime of 3 air changes per hour. However, this and similar studies normally only address a single infector in a single-person occupied space. In a realistic situation where there are multiple people exhaling CO_2_ and possibly only a handful of infected people present, the CO_2_ concentrations would not be expected to correlate to virus-laden aerosols in the same manner. Further studies are reviewed in our previous paper.^22^

Some field studies have monitored indoor CO_2_ concentration during the COVID-19 pandemic to assess ventilation effectiveness in a variety of building types and sometimes attempted to assess actual transmission risk based on CO_2_ concentrations. Our previous paper, Adzic et al.,^22^ reported on ventilation effectiveness in theatres measured using high-resolution CO_2_ monitoring. Vouriot et al.^23^ investigated the likelihood of transmission in school environments by monitoring CO_2_ concentration in 45 classrooms in the UK over several months, finding the risks to be higher in January compared with July. Tang et al.^24^ used CO_2_ as a proxy for SARS-CoV-2 transmission risk in an office and concluded that along with CO_2_ concentrations in the space, the occupants’ location and exposure time affects individual transmission risks, however, the uncertainty in their infection risk assessment was not quantified. Jones et al.^25^ developed a Relative Exposure Index (REI) which calculates the relative difference in inhaled dose of a comparator and reference scenario enabling an estimate of personal relative risk if sharing a scenario with an infector. As it is a relative metric, the uncertainties in infector viral emission rate cancel, but it does not give an indication of the absolute magnitude of the long-range transmission risk. Many of the risk-based models assume well-mixed spaces and therefore high-resolution CO_2_ monitoring in large spaces can be a valuable tool in identifying the distribution of risk.

There are limitations to attempts to link transmission risk based on ventilation rates directly to levels of CO_2_ concentrations and this is an intractable problem, however monitoring CO_2_ remains the most pragmatic and rapid method of assessing ventilation effectiveness in real-world occupied buildings and identifying spaces that pose a higher risk. The Scientific Advisory Group for Emergencies-Environmental Modelling Group (SAGE-EMG) and the Independent Scientific Pandemic Insights Group on Behaviours (SPI-B), both of which are scientific advisory groups for the UK Government, emphasised the importance of ventilation and CO_2_ monitoring during the pandemic and made a series of recommendations for guideline CO_2_ concentrations.^26,27^ Although not a direct quantitative indicator of SARS-CoV-2 transmission risk, CO_2_ is in exhaled breath and is an effective proxy for *occupancy relative to the levels of ventilation*, a parameter that does relate to the risk of long-range aerosol transmission in indoor spaces.^27^

In 2020, the UK Government suspended all culture, sports, music, and entertainment events.^28^ One year later, the UK Government ran the Events Research Programme (ERP). The ERP is, to date, the largest research programme of its kind worldwide.^29^ It aimed to explore how mass gathering events could reopen post-COVID-19 by conducting a series of pilot events from April to June 2021.^30^ Events were run under realistic pre-pandemic conditions, e.g., mask-wearing and physical distancing were not required. During the events, an evidence base was gathered to understand the influencing factors in SARS-CoV-2 transmission and how to minimise these. Other studies of events were conducted at similar times in other countries, but not on the same scale or with the same realistic conditions, i.e., most were “staged” events without normal attendees, or were retrospective studies (e.g. 2, 4). Examples include an indoor music concert in Germany which used a series of experimental “staged” events with three different hygiene and crowd movement strategies to examine the number of close contacts made between attendees, and CFD simulations to investigate the effect of ventilation on aerosol transmission.^31^ The study highlighted the importance of adequate ventilation, however, the actual indoor air quality during the three experimental events was not measured. An indoor music concert in Barcelona, Spain, was monitored in December 2020 and found that rapid antigen tests were a suitable SARS-CoV-2 transmission prevention strategy alongside adequate ventilation and mask-wearing by attendees.^32^

The ERP Environmental Study examined the risk of long-range airborne transmission indoors based on indicators of indoor air quality. CO_2_, an indicator of potentially poor ventilation, was monitored as a proxy for exhaled breath and exhaled aerosols that potentially contain virus particles, at numerous locations throughout the venues of the study. The study evaluated the ventilation relative to crowd density and long-range airborne transmission risks at ten indoor and hybrid indoor/outdoor venues of different types, functions, sizes, and layouts. The following paper and our previous papers^22,33^ present the methodology that was developed, for rapid, high-resolution measurement of indoor air quality (IAQ) during live mass gathering events in large venues and provides evidence-based information on some factors for building operators to consider, to limit the airborne transmission of SARS-CoV-2 and other airborne viruses. To the author’s knowledge, there was no previous published research around the world that presented significant IAQ data from mass gathering live events, during normal operations, so it had previously been difficult to create formal guidance on how venue ventilation schemes should be operated to maintain an acceptable level of transmission risk. The findings of the ERP Environmental study enabled such guidance to be produced for the sector and ultimately influenced Government policy around the scale and timing of reopening mass gathering events.^28,34^ Therefore, the UK Government ERP Environmental study represents a uniquely valuable field trial presenting evidence on IAQ during realistic building operations. This, and the rest of the evidence generated from the ERP, enabled most events to return to normal operations on 19 July 2021, having been closed since March 2020.

The following paper reports on the Environmental Study of the ERP. We begin by reviewing ventilation guidance for COVID mitigation, describe the methods of the study which include CO_2_ monitoring, classification of spaces and development of an IAQ Index with bands between A to G, and list all the events monitored by the study. We report results of the overall air quality at all events, the variability in different spaces around large venues, and identify types of spaces that are prone to poorer IAQ. We report on the impacts of occupancy, distribution of ventilation systems and discuss ventilation strategies found around the various venues, reporting results from settings such as theatres, nightclubs, sports, and music arenas.

## COVID-19 ventilation guidance

The risk of disease transmission is highly variable depending on the infectiousness of the infector, their aerosol-generating ability, aerosol generation activity, and the susceptibility of people sharing the space. However, evidence from past outbreaks and modelling work^25^ suggested that it is the very poorly ventilated spaces that are at greater risk, and those spaces that are ventilated to current UK building regulation standards pose a much lower risk of transmission and hence it may be more pragmatic to focus on improving those spaces that are at highest risk as the highest priority for interventions.^26,35^

Approved Document F^36^ of the building regulations and CIBSE^1^ Guide A^37^ provide a range of suggested airflows for various indoor space types that typically range from 5-10 L/s/person. These values originate from studies on occupant comfort to “stuffiness” (the general release of bioeffluents from occupants), where it has been determined that a high level of comfort in occupied spaces can be delivered with 8 L/s/person and therefore 10 L/s/person has often been chosen as a reasonable figure to provide occupant comfort, whilst balancing the energy requirements to deliver outside air. At the time of the ERP, many countries were recommending CO_2_ concentrations of 800–1000 ppm (equivalent to 10–12 L/s/person) as an appropriate target for ventilation rates. In the UK, guidance from SAGE-EMG and CIBSE recommended that spaces with CO_2_ consistently above 1500 ppm should be improved.^26,27^

During the early months of the COVID-19 pandemic CIBSE, REHVA, and ASHRAE released guidance to ensure that ventilation was improved in buildings as a means to reduce the risk of infection transmission by long-range aerosols.^38–40^ In particular, the CIBSE COVID-19 Ventilation Guidance version 1 and version 2 recommended maximising the ventilation rates but noting reasonable limits of occupant comfort.^41,42^ These first versions of the CIBSE COVID-19 Ventilation Guidance were written in the context of spring and summer of a temperate climate, and in October 2020, version 4 of the document adds more emphasis on reasonable over-ventilation considering occupant thermal discomfort, humidity, and energy use in preparation for the autumn and winter seasons.^43^ Version 4 recommended that the ventilation strategy should at least achieve the equivalent minimum ventilation rate for the space over the occupancy period as defined in current standards. This was the context of CIBSE guidance at the time of the ERP.

In July 2021, version 5 of CIBSE COVID-19 Ventilation guidance contained more detail on moderating energy demand and consideration of occupant comfort.^40^ As of September 2022, ASHRAE guidance also recommended that at least required minimum outdoor airflow rates for ventilation as specified by applicable codes and standards are provided and maintained,^44^ as well as increasing ventilation to 100% outdoor as conditions permit.^45^ REHVA version 4.1^46^ general advice is to supply as much outside air as reasonably possible, encouraging natural ventilation window opening regimes even if it results in occupant thermal discomfort. This version of the REHVA guidance has no specific mention of considering the energy penalty of overventilation. COVID-19 ventilation guidance documents that recommend minimum ventilation flow rates, as in current standards, enable building operators to utilise the most relevant guidance for their building scenarios. This could include recommended flow rates in CIBSE Guide A,^37^ ventilation guidance in EN16798,^47^ ASHRAE 62^48^ or local workplace regulations e.g., regulation 6 in Workplace (Health, Safety and Welfare) Regulations 1992.^49^ For England and Wales, the HSE has provided an approved code of practice and guidelines for meeting this requirement.^50^

## Methods

Exhaled breath accumulates due to insufficient ventilation, high occupancy of the space (whether temporarily or consistently), or sometimes both factors. Thus, CO_2_ concentrations indicate the fraction of the indoor air that has been exhaled by its occupants. Room steady-state concentrations increase with the number of occupants, their respiratory activity, and their body mass, and the rate of removal is solely dependent upon the ventilation rate. Hence, the risk of long-range airborne transmission was examined in this study, at numerous locations in different venues, based on *indicators* of indoor air quality (CO_2_), but without attempting to correlate risk *directly* to CO_2_ values as this direct correlation is impossible in the settings of live events with numerous occupants.

### Monitoring methods

The sensors used were Senseair Explora CO_2_, wireless, unobtrusive, battery-powered loggers that monitor concentrations of CO_2_, temperature (T), and relative humidity (RH). The sensors were installed in all identified indoor spaces in the venues and in some sheltered outdoor spaces. At a sampling interval of 2 min, the data logged was then encrypted and securely transmitted wirelessly via LoRaWAN (Long Range Wide Area Network) to a 4G hub from where data were sent to the cloud. The CO_2_ sensors in the loggers were non-dispersive infrared (NDIR), capable of measuring within a range of 400–5000 ppm at an accuracy of ±30 ppm (±3% of reading). These CO_2_ sensors have a built-in auto-calibration algorithm that tracks minimum CO_2_ values over eight-day intervals and compares them to a zero-point mark of 400 ppm. To ensure auto-calibration was reliable and there were no CO_2_ data drifts, sensor baseline values were checked at times when venues were unoccupied, at night, when CO_2_ readings are expected to match outdoor levels. The minimum CO_2_ values during events monitored were also checked for any unreliable data. The logged data was accessed and downloaded from an online database and also viewed in real-time on a dashboard. In total, 385 sensors were used in the study and the number of sensors deployed at each venue is shown in Table 2. At the Download Festival venue, the semi-outdoor nature of a large, circus-style tent meant that incoming rain could damage the sensors and there was no suitable place for the 4G hub to be connected to mains power. Therefore, battery-powered Hobo MX1102A sensors (also NDIR CO_2_ sensors with accuracy ±50 ppm/±5% which logged CO_2_, T and RH and stored data locally on the device) were used. These were calibrated in outdoor air, away from sources of CO_2_ such as busy roads, before installation and were otherwise operated identically to the Senseair sensors and their data were analysed in the same way.

Sensors were installed in numbers as appropriate to each venue and in consideration of the geometry of the venue spaces, practical restrictions on wall fittings, and the need to place them discreetly. Several loggers were placed in each space, on walls at a height of 1.6–2.3 m, away from vents, doors, or windows, and/or under auditoria seats, as appropriate. These heights were chosen so as to be within the breathing zone, assuming that occupants would be standing except in theatre auditoria, sports stadia seats, or in restaurants. The Institute for Air Quality Management and CIBSE have recommended sensors being placed within the breathing zone at heights of 1.1–1.7m.^51^ Sensor height of 1.5 m from the floor is suggested in the British Standard.^52^ The British Standard also suggests placing sensors 1–2 m from walls, although this was not possible in many venues as there were limited alternative fixing points aside from the backs of fixed chairs (in the theatre auditoria) and under guard rails (in the Crucible Theatre), or on walls where there was the risk of the sensors being tampered with by the attendees.

At the nightclub event, sensors were encased in cages with a large open grid to prevent accidental or intentional interference by a large and unstructured crowd. In some venues, sensors were placed higher from the floor to understand if stratification of air was occurring in the space and to measure CO_2_ concentration outside of the breathing zone, e.g., eight sensors were placed at 2.7 m at the Bramley Moore Dock Warehouse, 12 sensors were attached to the lighting gantries in the ACC auditorium, and two sensors were placed at 5.6 m, attached to speakers suspended from the roof in the Download Festival tent.

**Figure 1.**
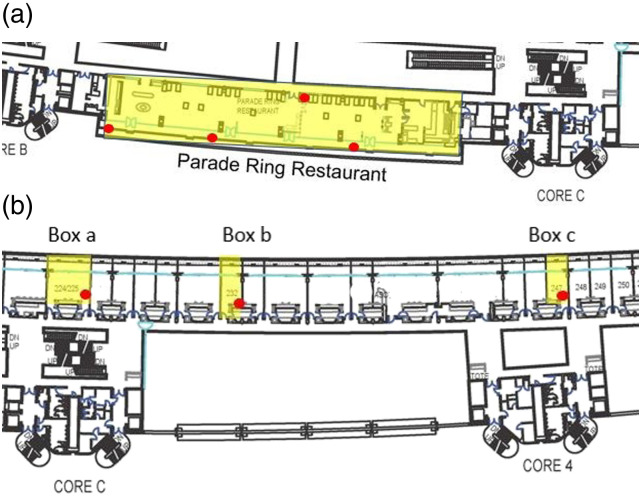
Installation of CO_2_ sensors at Ascot racecourse. Zones monitored are marked in yellow, and sensors are marked in red. (a) Parade Ring Restaurant (b) Private boxes.

The number of devices depended on the size of the room but as a minimum rule, 1–2 sensors were used in every space, to provide redundancy. Depending on the space size, either 1 to 4 sensors were placed, or up to 50 in a larger auditorium space, to detect the spatial variation of ventilation effectiveness at high-resolution. An example of this is shown in Figure 1, which depicts one typical installation at the Ascot Racecourse. The individual spaces monitored in this section of the venue, a restaurant and three private boxes, are highlighted in yellow and the sensors installed are marked on the drawing with red circles.

### Space classification

The first investigation found that ventilation strategies and space utilisation varied widely across the venues, and the majority of venues were observed to have a mixture of different spaces with different uses within them. For example, an outdoor stadium will typically have indoor concession stands, private boxes, bars, and toilets. Large venues have dedicated ingress and egress zones and these sometimes also serve as unstructured standing-only food and drink areas, or have shops and other concession stands in them, so some attendees may end up spending substantial time in various areas of the venue even though these are not officially designated as “spectator” zones. These types of spaces were often found to become “pinch point” areas of high concentration and restricted flows of both people and air, posing a higher risk for disease transmission from close contacts, droplet inhalation, and/or poor air quality with risk of aerosol transmission. Thus, all venues were divided into various spaces based on the space classification system described in Table 1 below, which classifies spaces based on ventilation strategy, and usage by attendees, where “structured” activities are more likely to involve allocated seating or event management (e.g. sports event with allocated seating, or theatre event), and “unstructured” events allow attendees to spend time throughout the venue as they prefer (e.g., race or music festival). In total, 179 individual spaces were monitored across 10 sports, music, and theatre venues, over 90 events (Table 2).

**Table 1.** Space Classification Criteria: (a) ventilation classification (b) use classification.

(a) Ventilation classification	(b) Use classification
• Outdoors	• Arrival and departure areas
• Outdoors, sheltered	• Dwelling areas
• Indoors, naturally ventilated, high ventilation	• Concessions/Bars-standing
• Indoors, naturally ventilated, low ventilation	• Bars/Restaurant-seated
• Indoors, mechanically ventilated	• Main activity areas (structured)
	• Main activity areas (unstructured)
	• Private boxes/meeting rooms
	• Toilets, corridors, lifts, stairwells (small, enclosed, short occupancy)

Mechanically ventilated systems were identified at the O2 Arena, the ACC Liverpool, The Grange festival auditorium, the Crucible Theatre, Piccadilly Theatre, and Leeds Playhouse, some of which had installed or recently improved modern mechanical ventilation systems in the spectator or social areas. Mechanical ventilation was also in use at some restaurants at the Ascot racecourse, and with some exceptions, in most of the indoor spaces and restaurants at Wembley Stadium. It should be noted that although all venues were allowed to operate at full capacity, this was not achieved during the ERP. Some naturally ventilated systems were also identified, at Ascot, Wembley Stadium, the Bramley Moore Dock Warehouse nightclub and Download Festival.

**Table 2.** Events and venues monitored by the environmental study during the three phases of the UK Government’s Events Research Programme.

Event	Venue	Date and phase	No. of events	No. of spaces	No. of sensors
World Snooker Championships	Crucible Theatre, Sheffield	Phase I 17/4/21–03/05/21	46	16	50
Emirates FA Cup semi-final	Wembley Stadium, London	Phase I 18/04/21	1	16	60
Carabao Cup final	Wembley Stadium, London	Phase I 25/04/21	1	20	71
Good Business Festival	ACC, Liverpool	Phase I 28/04/21	1	2	51
Circus presents ‘The First Dance’	Bramley Moore Dock Warehouse nightclub, Liverpool	Phase I 30/04/21–01/05/21	2	4	33
Brit Awards	The O2, London	Phase I 11/05/21	1	24	77
Emirates FA Cup final	Wembley Stadium, London	Phase I 15/05/21	1	26	85
EUROs 2020	Wembley Stadium, London	Phase II & III 13/06/21–11/07/21	8	26–31	76–85
Royal Ascot Races	Ascot Racecourse, Ascot	Phase II 15/06/21–19/06/21	5	31	70
Download Festival Pilot	Castle Donington, Donington Park	Phase II 18/06/21–20/06/21	3	3	19
Opera at the Grange festival	The Grange, Northington	Phase III 02/07/21–24/07/21	12	31	60
A Little Night Music	Playhouse Theatre, Leeds	Phase III 14/07/21–17/07/21	6	9	29
Comedy Nights	Piccadilly Theatre	Phase III 17/07/21–23/07/21	3	28	56

### Air quality classification and IAQ index

For the purpose of the ERP Environmental Study, it was assumed that all CO_2_ present indoors above a standard baseline outdoor value of 400 ppm has been exhaled by people in the space. An international standard on energy-efficient delivery of occupant comfort, BSEN16798,^47^ provided 4 levels of IAQ level of expectation based on occupancy (Table 3). The categories are related to the level of expectations the occupants may have. A normal level for an office space would be “Medium”. A higher level may be selected for occupants with special needs (children, elderly, persons with disabilities, etc.). A lower level will not provide any health risk but may decrease comfort and satisfaction with the space. Interestingly this standard is also most likely referring to the psychological discomfort of exposure to general bio-effluents from occupants, rather than the emission of pathogens from occupants, and neither standard considers the risk posed by poor ventilation, of exposure to indoor air pollutants emitted from building materials and furnishings, or to the increased risk of exposure to pathogens such as viruses and bacteria.

**Table 3.** Recommended targets for CO_2_ levels for Indoor Air Quality, adapted from BS EN 16798.

Category	Expectation of indoor environmental quality	CO_2_ above outdoors (ppm) assuming CO_2_ emission of 20 L/hr/person	Total indoor CO_2_ values (ppm)
I	High	550	950
II	Medium	800	1200
III	Moderate	1350	1750
IV	Low	1350	1750

Following from existing standards and COVID recommendations as outlined in *COVID-19 ventilation guidance* above and following SAGE-EMG recommendations, Table 4 presents the Indoor Air Quality Bands from A to G that were developed to form the basis for the analysis in this Environmental Study. For each space, an average performance was determined based on the average (mean) CO_2_ concentration for all events monitored in this space, where the CO_2_ concentrations were only considered during occupied times in the duration of an event, with the understanding that this represents performance under various typical occupancy scenarios. For each space, the maximum CO_2_ concentration recorded at all events was also identified, usually occurring during times of maximum occupancy. The spaces were then classified into Average and Maximum IAQ bands (Table 4).

**Table 4.** Classification for Air Quality bands from A to G used for the Events Research Programme.

Air quality bands	Classification	Range of CO_2_ concentrations - absolute values (ppm)	Range of excess CO_2_ concentrations - above outdoor (ppm)
At or marginally above outdoor levels	A	400–600	0–200
Target for enhanced aerosol generation (singing, aerobic activity)	B	600–800	200–400
Typical air quality design standards for offices	C	800–1000	400–600
Medium air quality	D	1000–1200	600–800
Design standard upper limits for most schools pre-COVID-19	E	1200–1500	800–1100
Priority for improvement (SAGE EMG)	F	1500–2000	1100–1600
Low ventilation/dense occupancy Must be prioritised for improvement	G	>2000	>1600

## Results

### Overall air quality across the board: ERP Phases I, II & III

**Figure 2.**
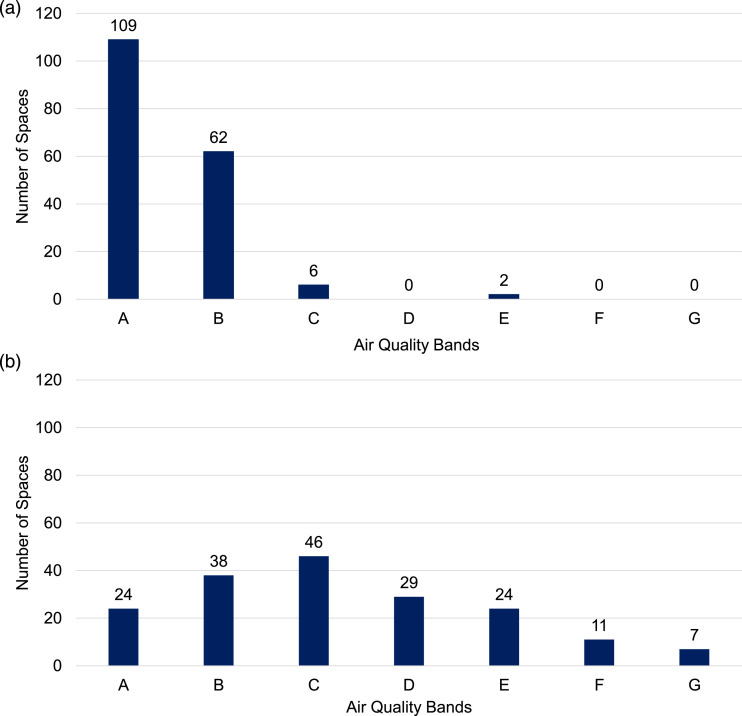
The number of spaces across the ten monitored venues aggregated by air quality bands: (a) Spatial and temporal Average and (b) Maximum CO_2_ values. Data includes all venues and events from ERP Phases I, II and III. (Figures reproduced following the published figures in^28^ following a full and final analysis of all events where data was collected).

For mitigation of COVID-19, indoor spaces where aerosol-generating activities occur (such as singing, aerobic activity or dancing) are encouraged to adopt a ventilation strategy capable of maintaining CO_2_ values at or below 800 ppm, or, in the ERP Air Quality bands A or B. Figure 2 shows the spatial CO_2_ average in given spaces calculated over the duration of events. The study finds that across the board, in Phases I, II and III of the ERP, average air quality was in bands A or B in 171 out of 179 monitored spaces, at almost all venues (Figure 2). Maximum CO_2_ values varied more than this: 10% of spaces were in air quality in bands F to G at peak times and at peak occupancies, the highest of which was found at a very large venue at around 75% occupancy. Time series of the data for these spaces revealed that these peak values were sometimes observed to persist for more than an hour or two, as will be discussed in the next sections.

### Air quality distribution in large venues across different spaces

Results from the O2 Arena and Wembley Stadium events held on 11 May and 15 May 2021 respectively, with occupancy of about 20% venue capacity, are presented below to illustrate the variation of average and maximum CO_2_ concentrations across a number of different spaces at the same event.

**Figure 3.**
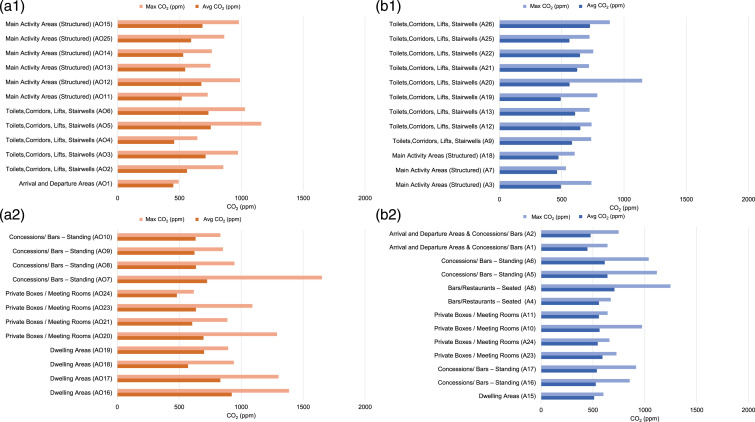
Average and Maximum CO_2_ values measured in two large venues (a1) and (a2) O2 Arena (b1) and (b2) Wembley Stadium.

The O2 Arena is a multi-purpose arena located in South-East London, inside the O2 Centre complex.

Spectator areas in the O2 Arena are the stalls, level 1 and level 2 seating. During the Brit Awards event, stalls were reserved for performing artists and stage installations, so spectators had access to level 1 and level 2 seating in the arena bowl, as well as concessions/bars in the arena concourse. The O2 has a capacity of 20,000, and 3,312 attendees attended the Brit Awards event. For the Brit Awards in the O2 Arena, 24 individual spaces were monitored, out of which the majority were indoors and mechanically ventilated.

Wembley Stadium is located in Northwest London and has a capacity of 90,000, with 18,720 spectators attending the FA Cup Final event on the 15 May. Spectator areas are stretched over five levels and are all outdoors. Although the main activity areas of Wembley Stadium are outdoors, the majority of spaces such as private boxes, restaurants, and bars are indoors, and their ventilation strategies vary depending on the space. For the FA Cup Final at Wembley Stadium, 20 individual spaces were monitored.

In both venues, spaces monitored included main activity areas (spectator seating), private boxes, standing and seated bars, concessions and restaurants, dwelling areas and toilets, lifts, and corridors. Figure 3 highlights the main activity areas where maximum CO_2_ concentrations never exceed 1000 ppm. This was expected at Wembley Stadium, as the main activity areas are outdoors in the stadium bowl and the average CO_2_ recorded is equivalent to the outdoor levels of approximately 400 ppm. The O2 Arena, although being an indoor space, had high ventilation rates in the seating area for the observed occupancy. However, in the O2 Arena, increased average, and maximum CO_2_ values were recorded in dwelling areas, bar concessions, private boxes and toilets and stairwells. Similar observations were made from monitoring Wembley Stadium bars, restaurants, boxes, and toilets.

### Types of spaces more prone to poor air quality

An example of spaces more prone to poor air quality are toilets, corridors, lifts, stairwells which are normally not designed for high ventilation rates as their occupancy is transient and low.

**Figure 4.**
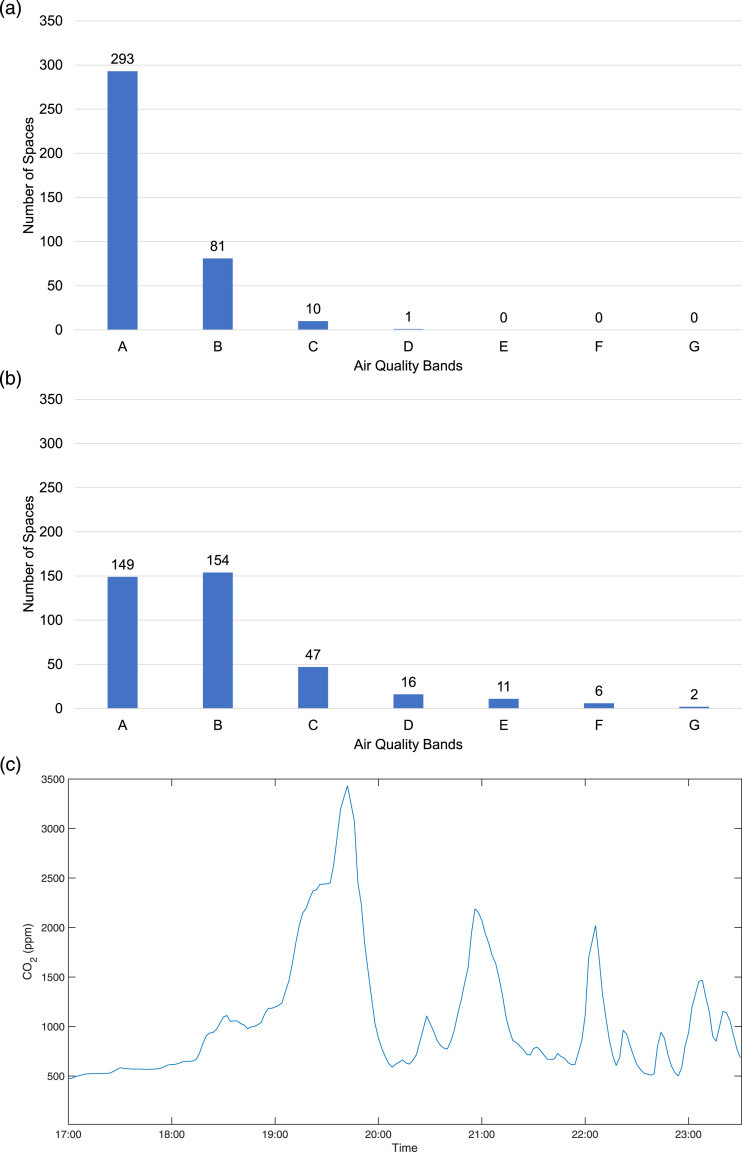
Monitored air quality in toilets, corridors, lifts, stairwells (small, enclosed, short occupancy) at all ERP events, separated into Air Quality bands. (a) Average CO_2_; (b) Maximum CO_2_; (c) CO_2_ Time-Series in a single toilet at a high capacity event at Wembley Stadium.

However, these spaces should be considered if long queues occur at events, as has been found to be the case: in particular around women’s toilets in the O2 Arena, as shown in Figure 3 (a1) above. In Figure 4 below, 23 toilets, corridors, lifts, and stairwell spaces monitored in six different venues over 72 events, are shown in aggregate (as 385 individual episodes). Spatial average and maximum air quality bands over the events duration were calculated and these are presented in terms of frequency of occurrence. The average air quality in these small, enclosed spaces is most frequently in A class (76%), but maximum air quality bands vary; these are most frequently in B class (40%) and are often in bands D and above. To understand this in further detail, an example is shown in Figure 4(c) of time-series for a single toilet space from a high occupancy (almost full capacity) event at Wembley Stadium in July 2021. The figure indicates very high concentrations of CO_2_ persisting for an hour at a time, with sharp peaks and steep decay rates. Moreover, CO_2_ values above 2000 ppm were observed for 46 minutes in total over the duration of the event. The longest continuous interval of CO_2_ above 2000 ppm was recorded just before the event started, and this lasted 36 minutes.

### The impact of occupancy, ventilation distribution and strategy

**Figure 5.**
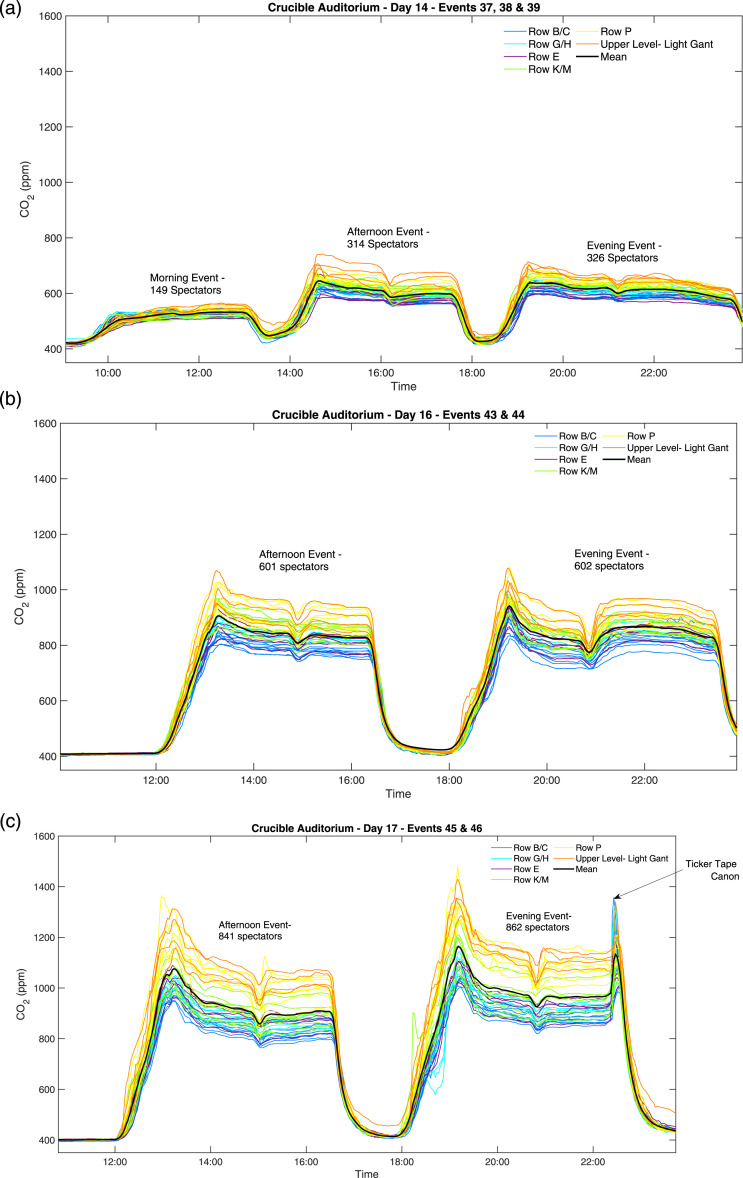
Ventilation distribution at a small theatre as observed from 44 CO_2_ sensors around the space at varying occupancies. The black line presents the spatial average in the auditorium (mean). (a), (b), and (c) are events held on different dates at different occupancies of, respectively: ∼30%, ∼60%, and ∼90%. Full occupancy at this theatre is 980 seats

Results from the Crucible show clearly the impact of increasing occupancy on the resulting CO_2_ (Figure 5). The low occupancy (30%) events given in Figure 5(a) show values of around 600 ppm. During higher occupancy (Figure 5(b)), the CO_2_ concentrations increase rapidly with the average peaking at >1000 ppm, then reducing to around 900 ppm as demand-controlled ventilation increases. However, the results demonstrate the space is not well mixed, with CO_2_ concentrations varying by nearly 400 ppm from the back row to the front of the auditorium. The back row of the theatre peaks at nearly 1400 ppm and stays above 1000 ppm for the entire event in high occupancy events given in Figure 5(c).

The combination of a less effective ventilation strategy and high crowd density (3,000 attendees per event but mostly clustered around a limited space within the event venue) at the Bramley Moore Dock Warehouse nightclub events contributed to the highest indoor CO_2_ concentrations observed in the Events Research Programme pilots in Phase I. The nightclub events were hosted in a unique venue: a 34,000 m^3^ Victorian-era warehouse. The building was naturally ventilated via six large warehouse door openings (49 m^2^) that were distributed along one side of the building at regular intervals. In one half of the building there was a bar, at the other end there was a dancefloor and stage. At the bar end, the three large openings were fully open. At the dance floor end, the openings were restricted by metal shutters and hanging vertical plastic strip curtains, “butchers screens”, to reduce noise egress from the venue. However, this endeavour lowered the supply of ventilation to the dancefloor. It was in this area that the crowd density was highest, due to clustering of people around the stage. The activities of the crowd: singing, dancing, shouting, etc., further increased the CO_2_ emission in this area compared to the more sedentary behaviour of those standing at the bar.

**Figure 6.**
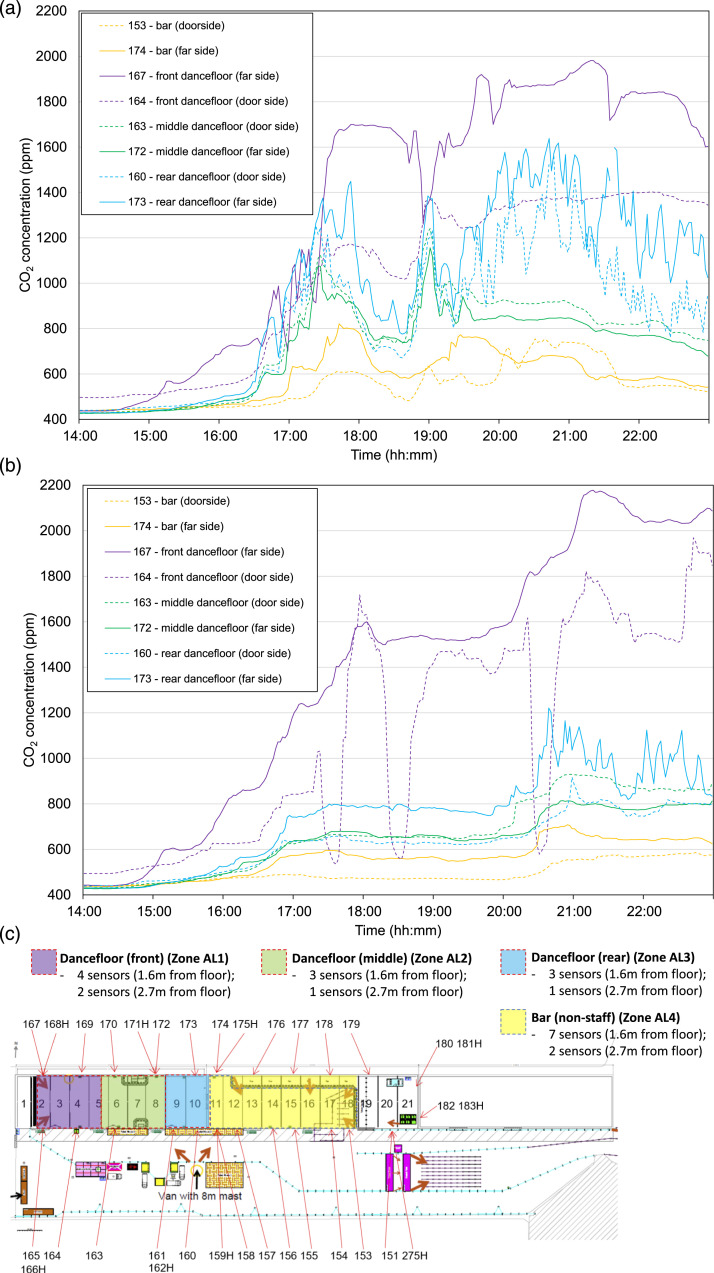
Measured CO_2_ concentration from a subset of sensors. Two sensors for each of the four zones. Dashed lines denote a sensor on the side of the building that has door openings. (a) Bramley Moore Dock Warehouse Nightclub Event 1; (b) Nightclub Event 2; (c) Nightclub monitored zones map with numbered sensors.

For air quality class analysis, the nightclub was divided into four zones: dancefloor (front), dancefloor (middle), dancefloor (rear), and bar (Figure 6(c)). The dancefloor (front) was classified as AQ band E, but the maximum values were in band G, compared to the air quality band target for ventilation of areas with enhanced aerosol generation of band B. It is evident that where an event attendee is located in the nightclub venue, modifies their exposure to exhaled breath. Those at the front, near the stage, were exposed to CO_2_ concentrations at times 1624 ppm higher than those in the bar area, indicating very poor ventilation, which significantly increases the risk of long-range airborne pathogen transmission.

Ventilation effectiveness, i.e., the ability for the incoming air to be homogeneously distributed within the indoor space, was identified as poor in the nightclub venue. By comparing the CO_2_ concentrations closest to and furthest from the ventilation openings (Figure 6, dashed lines versus solid lines) it can be observed that CO_2_ concentrations were generally lower closest to the door, especially in the area closest to the stage (dancefloor (front), compare sensor 164 and 167). This indicates that the incoming outdoor air was particularly poorly distributed further from the door area. The greatest difference was seen at the dancefloor (front) perhaps because this is the door people were most frequently using (and opening the butchers screens as they exited). This can also be seen at Event 2 (Figure 6(a)) where at 17:30, 18:30, and 20:30 (the points at which a DJ ended their set and a new one began) the CO_2_ concentrations in the area close to the door decreased as large numbers of people exited the venue to the outdoor area and in doing so both increased the size of the ventilation openings and reduced the CO_2_ emissions. Crucially, this reduction in CO_2_ concentration was not detected further from the door, which indicates that any airborne pathogens in this area would not be diluted or removed due to the poor ventilation effectiveness.

## Discussion

### IAQ index

The study developed a detailed IAQ Index that was used to classify spaces based on both average and maximum values of CO_2_. For this study, there was a need to rapidly investigate a large number of venues to provide an overall indication of performance at a wide set of venues that broadly represented the sector and identify the potential for long-range airborne transmission. The detailed IAQ Index allowed a nuanced understanding of the levels of exhaled breath that spectators or employees may be exposed to in different parts of venues over the entire duration of an event, and also to detect variability in the peak concentrations.

### Overall IAQ across the venues monitored

Overall, the spaces monitored had good average air quality in bands A or B, consistent with CO_2_ concentrations below 800 ppm and with very good ventilation for the spaces and the occupancies. The average air quality shown in Figure 2(a) was the spatial average air quality, which is also a temporal average during the entire event. Not all spaces were occupied at all times during the events, and this means that the averages do not present the full picture, hence maximum air quality bands helped identify spaces that had on occasion presented with high CO_2_ episodes that posed, temporarily, an increased risk of exposure to airborne diseases. It was found across the board that 18 of the 179 spaces presented maximum recorded CO_2_ concentrations above 1500 ppm, which indicated that in those spaces sometimes there was insufficient ventilation relative to the occupancy.

### Distribution of IAQ around spaces and implications for the evaluation of building performance in large buildings with multiple occupants

The approach to space classification and IAQ classification allowed the study to identify that average and maximum CO_2_ concentrations varied significantly: between venues, between types of events, between different events at the same venue run at different occupancies, and also among spaces within an event throughout a venue. This variability reflects the movement of occupants throughout a venue following event management patterns, as well as building design guidelines for different types of spaces. The variability in exposure within different locations at a single venue can be as significant as the variability between event types and venue types, leading to significant differences in exposure to exhaled breath and potentially to infection, amongst attendees within the same venue.

Localised ventilation strategies were very significant, especially in hybrid indoor-outdoor venues, and the nature, size, and organisation of an event are key to the outcomes in terms of IAQ throughout. This may be an important feature of other buildings with large occupancies that are in constant flux such as public transport hubs, schools, universities, and hospitals and would be worth further research.

### Types of spaces prone to poor IAQ and the value of space classification

A database of spaces was created for all venues and this allowed exploration of common types of spaces such as theatres or restaurants, which were investigated in depth outside the scope of this paper, and to identify types of spaces that are particularly prone to high CO_2_ concentrations. Such high concentrations were observed for prolonged periods in spaces such as toilets, corridors, lifts, and stairwells, as shown in Figure 4. These were found mainly in relation to high occupancy and high crowd density, which may have been outside of the original specification of the mechanical ventilation systems, even though these were most probably designed to deliver good ventilation performance on average for these spaces. In a minority of cases, not presented in this paper, high CO_2_ concentrations were observed where ventilation systems were found to be faulty or poorly maintained or where the ventilation strategy was not providing sufficient outdoor air. In these cases, feedback was provided to venue managers and was acted upon to provide immediate improvements.

### Ventilation effectiveness and impact of ventilation strategy

Several examples were found where the distribution of ventilation appeared less effective, for example, both in the Crucible auditorium (Figure 5), and in the nightclub (Figure 6), a distribution of CO_2_ can be noted, resulting in hot spots of poor IAQ even if average values throughout the venue were acceptable. This presents occupants of these venues with a very different experience with a wide range of potential comfort outcomes and a variable risk of exposure to airborne pathogens. Poor mixing of air cannot be observed if a low number of CO_2_ sensors is used, or if a CO_2_ sensor is only installed at the extract to control ventilation in a large space. Furthermore, the high resolution enabled the study to challenge the assumption that all indoor spaces are well-mixed with homogeneous indoor air quality.

### Impact of occupancy

Although the exact number of occupants in individual spaces is not analysed in this paper, venue and event operators provided insight into how the spaces were used during monitored events. This, and the field studies conducted by the research teams on site, helped quickly identify time intervals where individual spaces would most likely peak in occupancy to identify spaces that needed monitoring. In numerous settings, CO_2_ concentrations were found to closely follow patterns relating to event management and to relate to the local number of occupants and their distribution in time and space, as seen for example at the Crucible in Figure 5 and at the Wembley toilets via the time series in Figure 4(c). This presents an opportunity to improve local IAQ and reduce risks of transmission during future outbreaks by modifying event management strategies. This points to there being other available mitigations besides introducing costly and energy intensive modifications to ventilation systems. For example, time series from events where main activities occur indoors show a rapid drop in CO_2_ concentrations during intervals. Introducing more intervals where ventilation is not effective is recommended so that the space can be cleared of contaminants.

Event managers and venue operators should also have in mind that occupancy should be kept short in spaces prone to poor air quality such as toilets, lifts, and corridors. Long queues outside toilets at particular times during the events can increase the risk of both short- and long-range airborne transmission. Anecdotal evidence during the ERP showed that queues were sometimes deliberately created in order to prevent too many people from crowding in one space, but this strategy can backfire if the queue is simply displaced into another, even more risky space.

### High resolution monitoring

The method of high-resolution monitoring in space and time has proven to be very worthwhile and has generated valuable insight. The use of a large number of sensors enabled the study to identify spatial variations in CO_2_ within the venues, and thus comment on the ventilation effectiveness and variable exposure of occupants to exhaled breath in different parts of the same venue. The use of sensors that had been logging data at 2-min intervals and storing of the data on a central database also allowed for temporal changes in occupancy to be detected through the observed changes in CO_2_ concentration. Examples of this are during short breaks between theatre performances (intervals) as seen in Figure 5; or as can be seen in Figure 6, at the nightclub event as attendees exited between DJ sets and temporarily opened the door coverings, which were otherwise closed, introducing ventilation into the space.

Long-term monitoring may not be appropriate for every building and may not always be feasible due to the cost and lack of expertise of the occupants. Our study demonstrates that useful lessons can be learned from a rapid, temporary installation, and from monitoring carried out in real-world conditions and at realistic occupancy levels. These lessons can be used to inform venue building managers, operators and event managers quickly and robustly about areas that may need improvement in ventilation, and under which operational scenarios these improvements might be necessary.

### Limitations of the study

The monitoring and the subsequent analysis presented in this paper was intended to provide rapid evidence to the Events Research Programme for a representative selection of venues and events. The authors highlighted to the ERP the importance of monitoring a substantial selection of indoor auditoria and theatres, and the importance of understanding that “outdoor events” comprise many indoor spaces and that these must be monitored. Final case studies were selected by the ERP based on a wide variety of factors such as availability of venues and were not under the control of the authors.

The exact number of occupants in each space was not analysed for the purpose of this publication, although for some events these data are available. The total number of attendees at the events was used in the analysis to highlight the occupancy effect on CO_2_ concentrations (Figure 5), or to compare events of similar venue attendance as a percentage of capacity (Figure 3). Future work will focus on showing links between precise occupancy and IAQ as well.

It was assumed that all indoor CO_2_ concentrations recorded above average ambient levels (400 ppm) were attributed to human exhalation. On one occasion, however, in the Crucible Theatre, a “ticker tape” cannon was used at the end of the event at the winner announcement. This device uses CO_2_ as a propellant to eject confetti and temporarily increased the CO_2_ concentration. Thus, the exposure to exhaled breath in this instance may have been lower than the CO_2_ concentrations suggest in that short period. Theatrical smoke was also used at the Bramley Moore Dock Warehouse nightclub, however this did not artificially elevate the CO_2_ concentrations because a heated combination of glycol and water was used to create the smoke not dry ice.

Some events such as the nightclub, The Brit Awards, and the Piccadilly Theatre events, were run at significantly reduced occupancy. Conclusions cannot be made with certainty on what the air quality would be if events were to be run at full capacity. For example, the nightclub event was half full with around 3,000 attendees. It is likely that the air quality would be worse if the event was operated at full usual capacity of 6,000 attendees without any changes to the ventilation strategy.

### Ventilation guidelines: balancing energy and comfort with IAQ targets

Ventilation is a vital mitigation measure against COVID-19 transmission and the scale of the pandemic has only emphasised the need to ensure that the indoor built environment is designed and maintained with health outcomes in mind. Outside of the membership of CIBSE, there is less understanding in the wider community of what ventilation is, and how it is achieved effectively. Energy saving has rightly dominated targets for ventilation and building performance for many years, but the unintended consequences of this have resulted in increasingly airtight indoor spaces where ventilation and air conditioning systems are set to recirculate stale air, leakage from outdoors is minimised, and occupants have very little control of their environment or understanding of how it works.

The quality of ventilation across the entire UK building stock is not fully understood at present. There is growing evidence from a number of sectors that suggests that a wide range of building types may not always be adequately ventilated, especially in the winter months; this may be due to the operation, maintenance, design, or refurbishment and repurposing of existing buildings in operation. Additional consideration of the ventilation requirements in UK building regulations may be needed in future with a view to improve post-occupancy indoor air quality and build resilience to future infectious diseases. The COVID-19 pandemic has highlighted the vulnerability of the built environment to infectious disease transmission in indoor air via the long-range airborne route.

Initial COVID-19 ventilation guidance was focused on introducing as much outdoor air as possible into indoor environments and these have been updated over time to recommend that indoor spaces should not be over-ventilated in winter, as an energy-conscious approach with thermal comfort in mind, as well as sufficient outdoor air provided, is preferable. Our monitoring shows that most spaces had very good IAQ and in many situations there was potential to even lower ventilation rates, especially under reduced occupancy scenarios. To reduce energy costs and yet still improve performance for health, demand control ventilation and intelligent continuous monitoring of buildings may be reliable solutions.

## Conclusions

Results of the Environmental Study of the UK Government’s Events Research Programme, the largest IAQ monitoring programme worldwide of mass gathering venues during live events to date, were presented in this paper. A method was developed to rapidly deploy and measure CO_2_ concentrations, both spatially and temporally, at high resolution in each venue, to store data on an online database for further analysis, and to classify spaces based on usage and ventilation strategy, and determine their performance against an IAQ Index.

Overall, the spaces monitored had good air quality in bands A or B on average, consistent with CO_2_ values below 800 ppm and with very good ventilation for the spaces and the occupancies. Around 10% of spaces presented maximum recorded CO_2_ values above 1500 ppm, requiring improvement of ventilation for the occupancy in those spaces some of the time.

Some typical problem areas, with higher CO_2_ concentrations, were identified by the study, such as toilets, stairwells, and corridors, that may not have been designed for prolonged occupancy and are therefore more poorly ventilated, yet for disease transmission may become high risk areas when the occupancy is increased due to, e.g., long queues.

The high-resolution monitoring has highlighted some situations that led to variable and sometimes poor air quality in space and in time throughout the large venues. It revealed that for the purpose of infection control, ventilation strategies need to be based on realistic occupancy scenarios which include a variation of occupancy in time and space.

Although on the whole there was good to excellent IAQ at the events monitored, some spaces were under ventilated some of the time and some may have been overventilated for the occupancies, at the expense of increased energy demand if the space is heated or cooled. Future guidelines for ventilation may need to consider more intelligent and demand driven controls so that variations in occupancy and in ventilation targets can be catered for, with a view to balancing thermal comfort, ventilation, IAQ, and energy use in an optimal manner around the year.

## Note

a. Chartered Institution of Building Services Engineers.
